# New combinations in *Odontostemma* (Caryophyllaceae)

**DOI:** 10.3897/phytokeys.63.8181

**Published:** 2016-06-02

**Authors:** Richard K. Rabeler, Warren L. Wagner

**Affiliations:** 1 University of Michigan Herbarium – EEB, 3600 Varsity Drive, Ann Arbor, MI 48108-2228, USA; 2 Department of Botany, MRC-166, National Museum of Natural History, Smithsonian Institution, P.O. Box 37012, Washington, DC 20013-7012, USA

**Keywords:** Odontostemma, Arenaria, Caryophyllaceae, nomenclature

## Abstract

Sixty-three new combinations in *Odontostemma* (Alsineae, Caryophyllaceae) are made to accommodate placement of all currently recognized taxa of Arenaria subg. Odontostemma within the genus *Odontostemma*.

## Introduction

In their study of relationships in *Arenaria* L. and major lineages within the Alsinoideae using nuclear and plastid DNA, Harbaugh et al. (2010) found that three members of Arenaria subg. Odontostemma (Benth. ex G. Don) F.N. Williams clustered in a clade with *Pseudostellaria* Pax and *Lepyrodiclis* Fenzl, distinct from most of *Arenaria* s.s., instead clustering more closely with *Stellaria* L. and *Cerastium* L. This alignment was also seen in the broad study of family relationships by Greenberg and Donoghue (2011).

Sadeghian et al. (2015) completed the most recent and more comprehensive analyses of *Arenaria* in the broad sense. They confirmed the results of Harbaugh et al. (2010) and Greenberg and Donoghue (2011), showing that *Arenaria* s.s. and *Eremogone* Fenzl are in different clades and are each monophyletic and that Arenaria subg. Odontostemma is in a third clade separate from those including *Arenaria* s.s. and *Eremogone*; *Odontostemma* is also monophyletic. In addition, Arenaria subg. Solitaria McNeill is sister to *Odontostemma* and subg. *Dolophragma* (Fenzl) McNeill appears distantly related to any *Arenaria* species; both of these subgenera should also be excluded from *Arenaria* and treated as distinct genera.

With only seven names currently available in *Odontostemma* (six provided by Sadeghian et al. (2015) for species they sampled), nearly all members of Arenaria subg. Odontostemma require new combinations in *Odontostemma*. With active flora projects in India (Flora of India Checklist, in prep.) and China (Flora of China, e.g. Wu et al. 2001) bringing more information to light about these areas, we feel that it is time to supply the 63 combinations necessary for recognizing remaining taxa as members of the genus *Odontostemma*.

## Methods

The information about type specimens of the basionyms of the new combinations that we have included is based on examining protologues and searching major indices (Tropicos - http://www.tropicos.org/; JSTOR Global Plants - https://plants.jstor.org; Chinese Virtual Herbarium (CVH), http://www.cvh.org.cn), as well as websites of several individual herbaria (B [http://ww2.bgbm.org/herbarium/default.cfm], E [http://elmer.rbge.org.uk/bgbase/vherb/bgbasevherb.php], K [http://apps.kew.org/herbcat/navigator.do?_ga=1.234000091.472586779.1455676163], P [https://science.mnhn.fr/institution/mnhn/collection/p/item/search] , US [http://collections.nmnh.si.edu/search/botany/?ti=3], and WU [http://herbarium.univie.ac.at/database/search.php]) for extant specimens. We also examined images of specimens at A which had not been included in JSTOR and at KUN for twenty-one taxa where information in the CVH appeared to be incomplete. Herbarium abbreviations follow *Index Herbariorum* (Thiers 2016, continuously updated, http://sweetgum.nybg.org/science/ih/). We have examined a digital image from one (or more) of these sources for any specimen for which we cite a barcode in the type citations. In cases where specimen deposition is not clearly stated in a protologue, we have added “?” after the abbreviation where, based on information about the location of the herbarium where the author worked and/or deposited their herbaria (see Index of Botanists - http://kiki.huh.harvard.edu/databases/botanist_index.html), we expect, but cannot confirm, a type specimen should have been deposited.

In the cases where syntypes are cited, we have refrained from designating lectotypes. It is not a requirement for the names to be validly and effectively published and we consider those decisions should be made during the course of a taxon-level revision where a serious study of all specimens would lead to the best selections.

## Discussion

*Odontostemma* Benth. ex G. Don was originally described by Bentham to segregate *Odontostemma glandulosum* Benth. ex G. Don from *Arenaria* (Don 1831). Most subsequent authors have included *Odontostemma* in *Arenaria*, most recently as a subgenus (McNeill 1962, Wu et al. 2001). Arenaria subg. Odontostemma includes about 65 species (Wu et al. 2001), all native to eastern Asia with 57 species endemic to China (Wu et al. 2001). Wu et al. (2001) noted that species have sometimes been grouped into five sections (two of which have not been validly published); Sadeghian et al. (2015) suggested it is premature to suggest subdividing the genus pending additional taxon sampling.

*Odontostemma* taxa can be recognized by a combination of characters. Most members have two, or seldom three (or more in one species), styles, sepals that curve outward distally and are saccate proximally with truncate or acute apices, and seeds with an inflated testa that lacks reticulate striations. If one were to apply the generic segregations reported in Sadeghian et al. (2015) to the Wu et al. (2001) treatment of *Arenaria* in China, the 102 species would be distributed among five genera, with *Arenaria* comprising just six species. A key to these genera, based in part on McNeill (1962) and Wu et al. (2001), follows:

### Key to *Arenaria* and segregate genera

**Table:** 

1	Styles 2 (seldom 3; 4(5) in *Odontostemma weissianum*), capsule valves 4 (seldom 6; 8(10) in *Odontostemma weissianum*); plants diffuse	**2**
–	Styles 3, capsule valves 6 (rarely styles 2 and capsule valves 4, then plants densely pulvinate [some Arenaria subg. Dicranilla (Fenzl) F.N. Williams]); plants diffuse, caespitose, or pulvinate	**3**
2	Sepals often curved outward distally, often saccate proximally, obscurely veined, apex truncate or acute; seeds usually inflated, roughened, surface obscurely marked; east Asia	***Odontostemma***
–	Sepals straight, not saccate proximally, veins prominent, apex acute-acuminate or obtuse; seeds not inflated, surface often roughened and tuberculate; France [*Arenaria provincialis* Chater & G. Halliday]	***Arenaria*** (in part)
3	Plants densely caespitose or pulvinate	**4**
–	Plants diffuse, sometimes weakly caespitose	**6**
4	Sepals thickened proximally, veins 1-3, prominent	***Eremogone*** (in part)
–	Sepals not thickened proximally, veins indistinct	**5**
5	Plants densely caespitose but not pulvinate; cauline leaves sometimes overlapping, but not decussate; flowers solitary, rarely in pairs; sepal apex and margins hardened	***Solitaria***
–	Plants densely pulvinate (rarely densely caespitose); cauline leaves decussate in 4 rows; flowers solitary or in three-flowered cymes; sepal apex and margins scarious, but not hardened	***Dolophragma***
6	Leaves filiform to subulate, often grass-like, or short and needle-like with margins often white-scarious; sepals thickened proximally, veins often prominent	***Eremogone*** (in part)
–	Leaves linear to ovate to orbicular, neither grass-like nor needle-like, margins not scarious; sepals not or barely thickened proximally, veins obscure or prominent	***Arenaria*** (in part)

The name Odontostemma is a compound word formed from *odont*, Greek, tooth, and *stemma*, Greek, wreath or garland. Although Odontostemma appears to be feminine, the gender must be treated as neuter. According to Article 62.2 (see esp. 62.2(c)) of the International Code of Nomenclature (McNeill et al. 2012), a compound genus name takes the gender of the last element; -*stemma* is neuter.

This detail was apparently overlooked when five of the new combinations were made in Sadeghian et al. (2015). Article 32.2 of the International Code (McNeill et al. 2012) allows these names to be corrected “without change of the author citation or date”. These names should now be cited as follows: *Odontostemma barbatum* (Franch.) Sadeghian & Zarre, *Odontostemma ionandrum* (Diels) Sadeghian & Zarre, *Odontostemma pogonanthum* (W.W. Sm.) Sadeghian & Zarre, *Odontostemma roseiflorum* (Sprague) Sadeghian & Zarre, and *Odontostemma trichophorum* (Franch.) Sadeghian & Zarre.

## Taxonomic treatment

### Odontostemma

Taxon classificationPlantaeCaryophyllalesCaryophyllaceae

Benth. ex G. Don

Odontostemma Benth. ex G. Don, Gen. Hist. 1: 449. 1831
Arenaria subg. Odontostemma (Benth. ex G. Don) F.N. Williams, Bull. Herb. Boissier 3: 603. 1895.
Gooringia F.N. Williams, Bull. Herb. Boissier 5: 530. 1897.

#### Type.

*Odontostemma glandulosum* Benth. ex G. Don, Gen. Hist. 1: 449. 1831

#### Description.

Herbs annual, biennial, or perennial, diffuse, not pulvinate. Leaf blades linear to ovate, rarely subulate. Inflorescences cymose, terminal or sometimes axillary, the flowers often borne on long pedicels. Sepals often curved outward distally, often saccate, veins inconspicuous, margin membranous, apex truncate or acute. Petals usually longer than sepals (sometimes shorter, when cleistogamous flowers present), apex emarginate or shallowly bifid or toothed. Styles 2, seldom 3 [4(5) in *Odontostemma weissianum*]. Seeds often inflated, roughened, without reticulate striations.

About 65 species: Asia; 59 species (57 endemic) in China.

### New combinations

#### Odontostemma amdoense

Taxon classificationPlantaeCaryophyllalesCaryophyllaceae

(L.H. Zhou) Rabeler & W.L. Wagner comb. nov.

urn:lsid:ipni.org:names:77155278-1

Arenaria amdoensis L.H. Zhou in C.Y. Wu, Fl. Xizang. 1: 688. 1983.

##### Type.

China: Xizang: river beaches, Ando Xian, 4830 m, Qinghai-Xizang Freezing-soil Complex Exped. 551 (holotype, NWBI, not seen).

#### Odontostemma auricomum

Taxon classificationPlantaeCaryophyllalesCaryophyllaceae

(Y.W. Tsui ex L.H. Zhou) Rabeler & W.L. Wagner comb. nov.

urn:lsid:ipni.org:names:77155279-1

Arenaria auricoma Y.W. Tsui ex L.H. Zhou, Acta Biol. Plateau Sin. 6: 31. 1987.

##### Type.

China: Yunnan: mountain sands, Zhongdian, 4200–4750 m, 1 September 1962, Zhongdian Exped. 1831 (holotype KUN, KUN0510342; isotype, KUN, KUN1205404).

#### Odontostemma barbatum (Franch.) Sadeghian & Zarre var. hirsutissimum

Taxon classificationPlantaeCaryophyllalesCaryophyllaceae

(W.W. Sm.) Rabeler & W.L. Wagner comb. nov.

urn:lsid:ipni.org:names:77155332-1

Arenaria barbata var. hirsutissima W.W. Sm., Notes Roy. Bot. Gard. Edinburgh 11: 195. 1920.

##### Type.

China: Yunnan: dry open stony mountain meadows, eastern flank, Lijiang Range, 3350–3650 m, 27°40'N, August 1910, G. Forrest 6299 (holotype, E, E00313712; isotype, K, K000723902).

#### Odontostemma bomiense

Taxon classificationPlantaeCaryophyllalesCaryophyllaceae

(L.H. Zhou) Rabeler & W.L. Wagner comb. nov.

urn:lsid:ipni.org:names:77155280-1

Arenaria bomiensis L.H. Zhou in C.Y. Wu, Fl. Xizang. 1: 685. 1983.

##### Type.

China: Xizang: mountain grasslands, Bomi, ca. 3700 m, P.C. Tsoong 6347 (holotype, PE, not seen).

#### Odontostemma chamdoense

Taxon classificationPlantaeCaryophyllalesCaryophyllaceae

(C.Y. Wu ex L.H. Zhou) Rabeler & W.L. Wagner comb. nov.

urn:lsid:ipni.org:names:77155281-1

Arenaria chamdoensis C.Y. Wu ex L.H. Zhou in S.W. Liu, Fl. Qinghai. 1: 506. 1997.

##### Type.

China: Xizang: on gravel, Chamdo (Sheezha), 4600 m, 24 August 1976, C.Y. Wu 5033 (holotype, KUN, KUN1205408; isotype, KUN, KUN1207757).

#### Odontostemma delavayi

Taxon classificationPlantaeCaryophyllalesCaryophyllaceae

(Franch.) Rabeler & W.L. Wagner comb. nov.

urn:lsid:ipni.org:names:77155282-1

Arenaria delavayi Franch., Bull. Soc. Bot. France 33: 432. 1886.

##### Type.

China: Yunnan: in rock clefts near summit of Mt. Tsang-chan, above Tali, 4000 m, P.J.M. Delavay 1039 (holotype P, P01902938; isotypes: P [2 sheets], P01902939, P01902940).

#### Odontostemma dimorphitrichum

Taxon classificationPlantaeCaryophyllalesCaryophyllaceae

(C.Y. Wu ex L.H. Zhou) Rabeler & W.L. Wagner comb. nov.

urn:lsid:ipni.org:names:77155283-1

Arenaria dimorphitricha C.Y. Wu ex L.H. Zhou, Acta Biol. Plateau Sin. 6: 28. 1987.

##### Type.

China: Sichuan: margin of woods, grassy slope, Mu-li, Gi-bo-shan, 2800 m, 7 August 1937, F.I.B. Yunnan Exp., T.T. Yü 7644 (holotype, KUN, KUN1205413; isotype, A, A00235507).

#### Odontostemma dsharaense

Taxon classificationPlantaeCaryophyllalesCaryophyllaceae

(Pax & K. Hoffm.) Rabeler & W.L. Wagner comb. nov.

urn:lsid:ipni.org:names:77155284-1

Arenaria dsharaensis Pax & K. Hoffm. in Pax, Repert. Spec. Nov. Regni Veg. Beih. 12: 366. 1922.

##### Type.

China: East Tibet: along creeks near snow patches, Ta tsien lu, west chain of Dshara parallel, east Chung ku, 4700 m, H.W. Limpricht 1818 (holotype, WRSL?; isotype WU, WU0046569).

#### Odontostemma euodontum

Taxon classificationPlantaeCaryophyllalesCaryophyllaceae

(W.W. Sm.) Rabeler & W.L. Wagner comb. nov.

urn:lsid:ipni.org:names:77155285-1

Arenaria euodonta W.W. Sm., Notes Roy. Bot Gard. Edinburgh 11: 195. 1920.

##### Syntypes.

China: Yunnan: open situations on and amongst boulders, Kari Pass, Mekong-Yangtze Divide, 3960 m, 27°40'N, July 1914, G. Forrest 12891 (syntype, E, E00313702; isosyntypes, KUN, KUN0510655 [labeled “holotype”]), PE, PE00551428); China: SE Tibet: open stony pasture and screes, on Doker-La, Mekong-Salween Divide, 28°20'N, August 1917, G. Forrest 14640 (syntype, E, E00316014; isosyntype, K, K000723895).

#### Odontostemma filipes

Taxon classificationPlantaeCaryophyllalesCaryophyllaceae

(C.Y. Wu ex L.H. Zhou) Rabeler & W.L. Wagner comb. nov.

urn:lsid:ipni.org:names:77155286-1

Arenaria filipes C.Y. Wu ex L.H. Zhou, Acta Biol. Plateau Sin. 6: 34. 1987.

##### Type.

China: Sichuan: wooded margins, Mu-li, Gi-bo-shan, 2800 m, 7 August 1937, F.I.B. Yunnan Exp., T.T. Yü 7637 (holotype, KUN, KUN1205418; isotypes, A, A00235539, KUN, KUN1205415, PE [2 sheets], PE00551451, PE00551452).

#### Odontostemma fimbriatum

Taxon classificationPlantaeCaryophyllalesCaryophyllaceae

(Mattf.) Rabeler & W.L. Wagner comb. nov.

urn:lsid:ipni.org:names:77155333-1

Arenaria fimbriata Mattf., Notizbl. Bot. Gart. Berlin-Dahlem 11: 335. 1932; Cerastium fimbriatum E. Pritz., Bot. Jahrb. Syst. 29: 320. 1900, not Ledeb. (1815), nom. illeg.

##### Syntypes.

China: Sichuan?: highest regions, Tai pa Shan, August, Giraldi 1187 (B, presumably destroyed), Giraldi 1188 (B, presumably destroyed).

#### Odontostemma giraldii

Taxon classificationPlantaeCaryophyllalesCaryophyllaceae

(Diels) Rabeler & W.L. Wagner comb. nov.

urn:lsid:ipni.org:names:77155287-1

Lepyrodiclis giraldii Diels, Bot. Jahrb. Syst. 36 (Beibl. 82): 38. 1905; Arenaria giraldii (Diels) Mattf., Notizbl. Bot. Gart. Berlin-Dahlem 11: 336. 1932.

##### Type.

China: Shaanxi?: Im Ki shan, 65 km SE of Sce kin Tsuen, Giraldi 2599 (B, presumably destroyed).

#### Odontostemma inconspicuum

Taxon classificationPlantaeCaryophyllalesCaryophyllaceae

(Hand.-Mazz.) Rabeler & W.L. Wagner comb. nov.

urn:lsid:ipni.org:names:77155288-1

Arenaria inconspicua Hand.-Mazz., Symb. Sin. 7: 197. 1929.

##### Syntypes.

China: Yunnan: bare, muddy, mica and granite areas, Mekong-Salwin-Kette, back Pongatong, 28˚9'N, 4300-4375 m, 4 August 1916, H. Handel-Mazzetti 9677 (syntype, WU, WU0043550; isosyntype, K, K000723884); Doker-La, border of Tibet, 4600 m, 17 September 1915, H. Handel-Mazzetti 8146 (syntype, WU, WU0043551).

#### Odontostemma inornatum

Taxon classificationPlantaeCaryophyllalesCaryophyllaceae

(W.W. Sm.) Rabeler & W.L. Wagner comb. nov.

urn:lsid:ipni.org:names:77155289-1

Arenaria inornata W.W. Sm., Notes Roy. Bot. Gard. Edinburgh 11: 196. 1920.

##### Type.

China: Yunnan: on boulders and ledges of cliffs, Mekong-Salween Divide, 3650 m, 28°12'N, July 1917, G. Forrest 14444 (holotype, E, E00313710; isotype K, K000723894).

#### Odontostemma ionandrum (Diels) Sadeghian & Zarre var. melanotrichum

Taxon classificationPlantaeCaryophyllalesCaryophyllaceae

(H.F. Comber) Rabeler & W.L. Wagner comb. nov.

urn:lsid:ipni.org:names:77155335-1

Arenaria ionandra var. melanotricha H. F. Comber, Notes Roy. Bot. Gard. Edinburgh 18: 229. 1934.

##### Type.

China: Yunnan: moist chalky pasture, eastern flank of Lijiang Range, 27°35'N, 3350 m, September 1910, G. Forrest 6509, (holotype, E, E00313708; isotypes, ISBC0147277, KUN0511451).

#### Odontostemma karakorense

Taxon classificationPlantaeCaryophyllalesCaryophyllaceae

(Em. Schmid) Rabeler & W.L. Wagner comb. nov.

urn:lsid:ipni.org:names:77155336-1

Arenaria karakorensis Em. Schmid, Repert. Spec. Nov. Regni Veg. 31: 42. 1932.

##### Syntypes.

China: Xizang: Kiam, 5100 m, 11 August 1927, W. Bosshard s.n. (W, presumed destroyed) ; China: Aksai-Chin, ca. 5000 m, 5 September 1927, W. Bosshard s.n. (W, presumed destroyed).

#### Odontostemma leucasterium

Taxon classificationPlantaeCaryophyllalesCaryophyllaceae

(Mattf.) Rabeler & W.L. Wagner comb. nov.

urn:lsid:ipni.org:names:77155290-1

Arenaria leucasteria Mattf., Notizbl. Bot. Gart. Berlin-Dahlem 11: 334. 1932.

##### Type.

China: Szechuan: in scree, Mt. Konka, Risonquemba, Konkaling, 5080 m, June-August 1928, J.F. Rock 16847 (holotype, B, presumed destroyed; isotypes, E, E00313707, GH, GH00053922, N [not seen], US, US00103300).

#### Odontostemma littledalei

Taxon classificationPlantaeCaryophyllalesCaryophyllaceae

(Hemsl.) Rabeler & W.L. Wagner comb. nov.

urn:lsid:ipni.org:names:77155291-1

Arenaria littledalei Hemsl., Bull. Misc. Inform. Kew 1896: 209. 1896. Gooringia littledalei (Hemsley) F.N. Williams, Bull. Herb. Boissier 5: 530. 1897.

##### Type.

China: Tibet, Goring Valley, 30°12'N, 90°25'W, 5000 m; St. George R. Littledale s.n. (holotype, K, K000723881).

#### Odontostemma longicaule

Taxon classificationPlantaeCaryophyllalesCaryophyllaceae

(C.Y. Wu ex L.H. Zhou) Rabeler & W.L. Wagner comb. nov.

urn:lsid:ipni.org:names:77155292-1

Arenaria longicaulis C.Y. Wu ex L.H. Zhou, Acta Biol. Plateau Sin. 6: 38. 1987.

##### Type.

China: Yunnan: 1938, Nat. Pek. Univ. Exped. s.n. (holotype, PE, not seen).

#### Odontostemma longipes

Taxon classificationPlantaeCaryophyllalesCaryophyllaceae

(C.Y. Wu ex L.H. Zhou) Rabeler & W.L. Wagner comb. nov.

urn:lsid:ipni.org:names:77155293-1

Arenaria longipes C.Y. Wu ex L.H. Zhou, Acta Biol. Plateau Sin. 6: 39. 1987.

##### Type.

China: Sichuan: cliffs, Muli, Shao-siang-liang-tze, ca. 3500 m, 16 August 1937, F.I.B. Yunnan Exp., T.T. Yü 7743 (holotype, KUN, KUN1205431; isotypes, A, A00235717, KUN, KUN0510918, PE [2 sheets], PE00024087, PE00551827).

#### Odontostemma longipetiolatum

Taxon classificationPlantaeCaryophyllalesCaryophyllaceae

(C.Y. Wu ex L.H. Zhou) Rabeler & W.L. Wagner comb. nov.

urn:lsid:ipni.org:names:77155294-1

Arenaria longipetiolata C.Y. Wu ex L.H. Zhou, Acta Biol. Plateau Sin. 6: 29. 1987.

##### Type.

China: Sichuan: forests, Mu-li, Between Gei-bao-shan and Mu-li-si, 2580 m [2800 m on holotype, transcription error?], 7 August 1937, F.I.B. Yunnan Exp., T.T. Yü 7655 (holotype, KUN, KUN1205433; isotypes, IBSC, IBSC0147267, KUN, KUN1205434, PE [2 sheets], PE00551828, PE 00551829).

#### Odontostemma longisetum

Taxon classificationPlantaeCaryophyllalesCaryophyllaceae

(C.Y. Wu) Rabeler & W.L. Wagner comb. nov.

urn:lsid:ipni.org:names:77155295-1

Arenaria longiseta C.Y. Wu in C.Y. Wu et al., Fl. Yunnan. 6: 835. 1995.

##### Type.

China: Yunnan: Deqen, 3800-3900 m, 13 August 1940, K.M. Feng 6599 (holotype, KUN, KUN1205436; isotypes, KUN [2 sheets], KUN1205435, KUN1207758).

#### Odontostemma longistylum

Taxon classificationPlantaeCaryophyllalesCaryophyllaceae

(Franch.) Rabeler & W.L. Wagner comb. nov.

urn:lsid:ipni.org:names:77155337-1

Arenaria longistyla Franch., Bull. Soc. Bot. France 33: 433. 1886.

##### Type.

China: Yunnan: on Mt. Li-kiang, 4000 m, 13 August 1886, P.J.M. Delavay 2103 (holotype, P, P04966773).

#### Odontostemma longistylum var. pleurogynoides

Taxon classificationPlantaeCaryophyllalesCaryophyllaceae

(Diels) Rabeler & W.L. Wagner comb. nov.

urn:lsid:ipni.org:names:77155339-1

Arenaria longistyla var. pleurogynoides Diels, Notes Roy. Bot. Gard. Edinburgh 5: 182. 1912.

##### Type.

China: Yunnan: on limestone drift at base of cliffs, eastern flank of Lijiang Range, 27˚20'N, 3650 m, September 1906, G. Forrest 2902 (holotype, E, E00313706; isotype, P, P04966776).

#### Odontostemma mairei

Taxon classificationPlantaeCaryophyllalesCaryophyllaceae

(H. Lév.) Rabeler & W.L. Wagner comb. nov.

urn:lsid:ipni.org:names:77155296-1

Cerastium mairei H. Lév., Repert. Spec. Nov. Regni Veg. 13: 341. 1914, not Arenaria mairei Emb. (1933); Arenaria iochanensis C.Y. Wu in C. L. Tang, Fl. Reipubl. Popularis Sin. 26: 241. 1996.

##### Type.

China: Yunnan: on rocks, du lo-Chan summit, 3400 m, August 1913, E. E. Maire s.n. (holotype, E, E00313695).

#### Odontostemma melanandrum

Taxon classificationPlantaeCaryophyllalesCaryophyllaceae

(Maxim.) Rabeler & W.L. Wagner comb. nov.

urn:lsid:ipni.org:names:77155297-1

Cerastium melanandrum Maxim., Bull. Acad. Imp. Sci. Saint-Pétersbourg, Sér. 3, 26: 429. 1880; Arenaria melanandra (Maxim.) Mattf. ex Hand.-Mazz., Symb. Sin. 7: 202. 1929.

##### Type.

China: Kansu, 1873, Przewalski s.n. (LE?).

#### Odontostemma melandryiforme

Taxon classificationPlantaeCaryophyllalesCaryophyllaceae

(F.N. Williams) Rabeler & W.L. Wagner comb. nov.

urn:lsid:ipni.org:names:77155298-1

Arenaria melandryiformis F.N. Williams, J. Linn. Soc., Bot. 38. 399. 1909.

##### Syntypes.

China: Xizang, Beong-chin, W of Chumbi, 1882, King’s collector 1127 (K, K000742175); China: Xizang, Syam-poo, Chumbi district, 1884, King’s collector 123 (K, K000742176).

#### Odontostemma melandryoides

Taxon classificationPlantaeCaryophyllalesCaryophyllaceae

(Edgew.) Rabeler & W.L. Wagner comb. nov.

urn:lsid:ipni.org:names:77155299-1

Arenaria melandryoides Edgew. in Hook.f., Fl. Brit. India 1: 241. 1874.

##### Type.

India: Sikkim, alt. 4260-5480 m, J. D. Hooker 13 (holotype, K?, not seen; isotype, GH, GH00037640).

#### Odontostemma membranisepalum

Taxon classificationPlantaeCaryophyllalesCaryophyllaceae

(C.Y. Wu) Rabeler & W.L. Wagner comb. nov.

urn:lsid:ipni.org:names:77155300-1

Arenaria membranisepala C.Y. Wu in C.Y. Wu et al., Fl. Yunnan. 6: 155, Addenda 835. 1995.

##### Type.

China: Yunnan: Lijiang?, C.Y. Wu & D.Y. Leu 21238 (holotype, KUN, KUN1205438).

#### Odontostemma minimum

Taxon classificationPlantaeCaryophyllalesCaryophyllaceae

(C.Y. Wu ex L.H. Zhou) Rabeler & W.L. Wagner comb. nov.

urn:lsid:ipni.org:names:77155301-1

Arenaria minima C.Y. Wu ex L.H. Zhou, Acta Biol. Plateau Sin. 6: 35. 1987.

##### Type.

China: Sichuan: mountain rock slope, Muli, Wachin, Jin-chang, 4000 m, 27 October 1937, F.I.B. Yunnan Exp., T.T. Yü 14649 (holotype: KUN, KUN0510991).

#### Odontostemma monanthum

Taxon classificationPlantaeCaryophyllalesCaryophyllaceae

(F. N. Williams) Rabeler & W.L. Wagner comb. nov.

urn:lsid:ipni.org:names:77155302-1

Arenaria monantha F.N. Williams, J. Linn. Soc., Bot. 38: 401. 1909.

##### Type.

China: Xizang: hills above Lhassa, August 1904, H.J. Walton 1138 (holotype, K, K000723880; isotype, BM, BM000946346).

#### Odontostemma moniliferum

Taxon classificationPlantaeCaryophyllalesCaryophyllaceae

(Mattf.) Rabeler & W.L. Wagner comb. nov.

urn:lsid:ipni.org:names:77155303-1

Arenaria monilifera Mattf., Notizbl. Bot. Gart. Berlin-Dahlem 11: 334. 1932.

##### Type.

China: Xizang: Tongolo, 17 June 1894, J.A. Soulié 2507b (holotype, B, presumed destroyed).

#### Odontostemma napuligerum

Taxon classificationPlantaeCaryophyllalesCaryophyllaceae

(Franch.) Rabeler & W.L. Wagner comb. nov.

urn:lsid:ipni.org:names:77155304-1

Arenaria napuligera Franch., Bull. Soc. Bot. France 33: 429. 1886.

##### Syntypes.

China: Yunnan, in rock clefts, Mt. Koua-la-po, near Hokim, 26 April 1884, P. J. M. Delavay 87 (syntypes, P [3 sheets], P01902952, P01902953, P01902954; isosyntypes, K, K000723890, KUN, KUN0511009); along rocky road to Yen-tze-hay, near Lankong, 2500 m, P. J. M. Delavay 1665 (syntypes, P [3 sheets], P01902955, P01902956, P01902957; isosyntype, K, K000723891).

#### Odontostemma napuligerum var. monocephalum

Taxon classificationPlantaeCaryophyllalesCaryophyllaceae

(W.W. Sm.) Rabeler & W.L. Wagner comb. nov.

urn:lsid:ipni.org:names:77155341-1

Arenaria napuligera var. monocephala W.W. Sm., Notes Roy. Bot. Gard. Edinburgh 11: 196. 1920.

##### Type.

China: Xizang: at Ka-gwr-pw temple, near the Yunnan frontier, in alpine turf on precipices, 4725 m, July 1913, F.K. Ward 814 (holotype, E, E00313701).

#### Odontostemma nigricans

Taxon classificationPlantaeCaryophyllalesCaryophyllaceae

(Hand.-Mazz.) Rabeler & W.L. Wagner comb. nov.

urn:lsid:ipni.org:names:77155305-1

Arenaria nigricans Hand.-Mazz., Symb. Sin. 7: 196. 1929.

##### Type.

China: Yunnan: between saddles of Mt. Lamatso, between Yungning and Dschungdien (Zhongdian?), dry limestone cliffs, 3200 m, 12 August 1915, H. Handel-Mazzetti 7609 (holotype, WU, WU0043552; isotype, E, E00313704).

#### Odontostemma nigricans var. zhenkangense

Taxon classificationPlantaeCaryophyllalesCaryophyllaceae

(C.Y. Wu ex L.H. Zhou) Rabeler & W.L. Wagner comb. nov.

urn:lsid:ipni.org:names:77155342-1

Arenaria zhenkangensis C.Y. Wu ex L.H. Zhou, Acta Biol. Plateau Sin. 6: 36. 1987; Arenaria nigricans var. zhenkangensis (C.Y. Wu ex L.H. Zhou) C.Y. Wu in C. L. Tang, Fl. Reipubl. Popularis Sin. 26: 209. 1996.

##### Type.

China: Yunnan: on rocky slope, Zhengkang, Snow Range, Hsiaoshiishan, 2850 m [2500 m on holotype (transcription error?), 2 August 1938, F.I.B. Yunnan Exp., T.T. Yü 17133 (holotype, KUN, KUN1205466; isotypes, KUN, KUN1205463, PE, PE00024079).

#### Odontostemma nivalomontanum

Taxon classificationPlantaeCaryophyllalesCaryophyllaceae

(C.Y. Wu ex L.H. Zhou) Rabeler & W.L. Wagner comb. nov.

urn:lsid:ipni.org:names:77155306-1

Arenaria nivalomontana C.Y. Wu ex L.H. Zhou, Acta Biol. Plateau Sin. 6: 29. 1987.

##### Type.

China: Yunnan: on rocky slope, Zhangkang, Xueshang, 2900 m, 31 July 1938, F.I.B. Yunnan Exp., T.T. Yü 17125 (holotype, KUN, KUN0511016); isotypes, PE [2 sheets], PE00551915, PE00551916).

#### Odontostemma omeiense

Taxon classificationPlantaeCaryophyllalesCaryophyllaceae

(C.Y. Wu ex L.H. Zhou) Rabeler & W.L. Wagner comb. nov.

urn:lsid:ipni.org:names:77155307-1

Arenaria omeiensis C.Y. Wu ex L.H. Zhou, Acta Biol. Plateau Sin. 6: 30. 1987.

##### Type.

China: Sichuan: under thickets, Omei-hsien, Mt. Omei, ca. 3100 m, 5 August 1938, W.P. Fang 12979 (holotype, KUN, KUN0511017; isotype, A00235605]).

#### Odontostemma paramelanandrum

Taxon classificationPlantaeCaryophyllalesCaryophyllaceae

(H. Hara) Rabeler & W.L. Wagner comb. nov.

urn:lsid:ipni.org:names:77155308-1

Arenaria paramelanandra H. Hara, J. Jap. Bot. 52: 193. 1977.

##### Type.

Nepal: Chakure Lekh, S of Jumla, 14000 ft, 21 July 1952, O. Polunin, W.R. Sykes, & L.H.J. Williams 4827 (holotype, BM, BM000521527) .

#### Odontostemma pharense

Taxon classificationPlantaeCaryophyllalesCaryophyllaceae

(McNeill & Majumdar) Rabeler & W.L. Wagner comb. nov.

urn:lsid:ipni.org:names:77155309-1

Arenaria pharensis McNeill & Majumdar, Bot. J. Linn. Soc. 80: 373. 1980.

##### Type.

China: Xizang: Phari plain, 4360 m, 14 October 1928, J. C. Dawa 398 (holotype, CAL, photo of holotype, DAO; isotype K, K000742178, photo of isotype, DAO).

#### Odontostemma polyspermum

Taxon classificationPlantaeCaryophyllalesCaryophyllaceae

(C.Y. Wu ex L.H. Zhou) Rabeler & W.L. Wagner comb. nov.

urn:lsid:ipni.org:names:77155310-1

Arenaria polysperma C.Y. Wu ex L.H. Zhou, Acta Biol. Plateau Sin. 6: 33. 1987.

##### Type.

China: Yunnan: side of mountain stream, Upper Kiukiang Valley, (Clulung) Chialahmuto, 3700 m, 7 August 1938, F.I.B. Yunnan Exp., T.T. Yü 19742, (holotype, KUN, KUN1205439; isotypes, A, A00235512, PE [3 sheets], PE00551959, PE00551960, PE00551961).

#### Odontostemma pseudostellaria

Taxon classificationPlantaeCaryophyllalesCaryophyllaceae

(C.Y. Wu, L.H. Zhou & W.L. Wagner) Rabeler & W.L. Wagner comb. nov.

urn:lsid:ipni.org:names:77155311-1

Arenaria pseudostellaria C.Y. Wu, L.H. Zhou & W.L. Wagner, Fl. China 6: 59. 2001. Arenaria linearifolia Franch., Pl. Delavay. 97. 1889, not Poiret (1804), nor Desvaux (1816); Moehringia linearifolia (Franch.) F.N. Williams, J. Linn. Soc., Bot. 34: 437. 1899; Moehringella linearifolia (Franch.) H. Neumayer, Verh. Zool.-Bot. Ges. Wien 73: 14. 1923.

##### Type.

China: Yunnan: in woods, Fang-yang-tchang, above Mo-so-yn, 3000 m, 17 June 1887, P.J.M. Delavay 2899 (holotype, P, P04987073).

#### Odontostemma quadridentatum

Taxon classificationPlantaeCaryophyllalesCaryophyllaceae

(Maxim.) Rabeler & W.L. Wagner comb. nov.

urn:lsid:ipni.org:names:77155312-1

Lepyrodiclis quadridentata Maxim., Fl. Tangut. 84. 1889; Arenaria quadridentata (Maxim.) F.N. Williams, J. Linn. Soc., Bot. 33: 432. 1898.

##### Type.

China: Qinghai: Danube-ngvarsi valley, 16 May 1885, G.N. Potanin s.n. (LE, not seen).

#### Odontostemma reductum

Taxon classificationPlantaeCaryophyllalesCaryophyllaceae

(Hand.-Mazz.) Rabeler & W.L. Wagner comb. nov.

urn:lsid:ipni.org:names:77155313-1

Arenaria reducta Hand.-Mazz., Kaiserl. Akad. Wiss. Wien, Math.-Naturwiss. Kl., Anz. 57: 47. 1920.

##### Type.

China: Yunnan: alpine meadows on the W side of Mt. Piepun, SE of Dschungdien, 4400-4650 m, 11 August 1914, H. Handel-Mazzetti, 4725 (holotype, WU, WU0043553).

#### Odontostemma rockii

Taxon classificationPlantaeCaryophyllalesCaryophyllaceae

(Diels) Rabeler & W.L. Wagner comb. nov.

urn:lsid:ipni.org:names:77155314-1

Arenaria rockii Diels, Notizbl. Bot. Gart. Berlin-Dahlem 9: 1027. 1926.

##### Type.

China: Yunnan: in limestone gravel, eastern slopes of Mount Dyinaloko, northern peak of the Likiang Snow Range, 4725 m, August 1923, J.F. Rock 10398 (holotype, B, presumably destroyed; isotype, US, US00103309).

#### Odontostemma saginoides

Taxon classificationPlantaeCaryophyllalesCaryophyllaceae

(Maxim.) Rabeler & W.L. Wagner comb. nov.

urn:lsid:ipni.org:names:77155343-1

Arenaria saginoides Maxim., Fl. Tangut. 89. 1889.

##### Type.

China: northeastern Tibet, on rocks, 3960 m, 9 July 1884, C.J. Maximowicz? (LE?, not seen).

#### Odontostemma salweenense

Taxon classificationPlantaeCaryophyllalesCaryophyllaceae

(W.W. Sm.) Rabeler & W.L. Wagner comb. nov.

urn:lsid:ipni.org:names:77155315-1

Arenaria salweenensis W.W. Sm., Notes Roy. Bot. Gard. Edinburgh 12: 194. 1920.

##### Type.

China: Yunnan: on stony pasture and humus-covered boulders in side valleys, Maikha-Salween Divide, 26º25'N, 3050 m, August 1919, G. Forrest 18474 (holotype, E [mounted on 2 sheets], E00117725 & E00117578; isotypes, K, K000723893, KUN, KUN0511179, WSY [2 sheets], WSY0062587, WSY0095442).

#### Odontostemma schneiderianum

Taxon classificationPlantaeCaryophyllalesCaryophyllaceae

(Hand.-Mazz.) Rabeler & W.L. Wagner comb. nov.

urn:lsid:ipni.org:names:77155316-1

Arenaria schneideriana Hand.-Mazz., Kaiserl. Akad. Wiss. Wien, Math.-Naturwiss. Kl., Anz. 57: 46. 1920.

##### Type.

China: Yunnan: in gravelly limestone, W side of Mt. Piepun, SE of Dschungdien (Zhongdian?), 4400-4700 m, 11 August 1914, H. Handel-Mazzetti, 4724 (holotype, WU, WU0043554; isotypes, E, E00313696, KUN, KUN0511180).

#### Odontostemma setiferum

Taxon classificationPlantaeCaryophyllalesCaryophyllaceae

(C.Y. Wu ex L.H. Zhou) Rabeler & W.L. Wagner comb. nov.

urn:lsid:ipni.org:names:77155317-1

Arenaria setifera C.Y. Wu ex L.H. Zhou, Acta Biol. Plateau Sin. 6: 37. 1987.

##### Type.

China: Yunnan: Bijiang, open slope, Chih-tse-lo, 4000 m, 4 September 1933, H.T. Tsai 54133 (holotype, KUN, KUN0511301; isotype, A, A00235713).

#### Odontostemma spathulifolium

Taxon classificationPlantaeCaryophyllalesCaryophyllaceae

(C.Y. Wu ex L.H. Zhou) Rabeler & W.L. Wagner comb. nov.

urn:lsid:ipni.org:names:77155318-1

Arenaria spathulifolia C.Y. Wu ex L.H. Zhou, Acta Biol. Plateau Sin. 6: 35. 1987.

##### Type.

China: Yunnan: open hillside, N flank of Haba Snow Range, 3500-4200 m, 20 August 1939, K.M. Feng 2082 (holotype, KUN, KUN0511307; isotypes, A, A00235684, KUN, KUN1205447).

#### Odontostemma szechuense

Taxon classificationPlantaeCaryophyllalesCaryophyllaceae

(F.N. Williams) Rabeler & W.L. Wagner comb. nov.

urn:lsid:ipni.org:names:77155344-1

Arenaria szechuensis F.N. Williams, J. Linn. Soc., Bot. 34: 437. 1899.

##### Type.

China: Sichuan: Tachien-lu, 1893, J.A. Soulié 814 (holotype, K, K000723892).

#### Odontostemma thangoense

Taxon classificationPlantaeCaryophyllalesCaryophyllaceae

(W.W. Sm.) Rabeler & W.L. Wagner comb. nov.

urn:lsid:ipni.org:names:77155319-1

Arenaria thangoensis W.W. Sm., Rec. Bot. Surv. India 4: 180. 1911.

##### Type.

India: Thango, 13000-14000 ft.; W.W. Smith 2572 (E?, not seen).

#### Odontostemma trichophyllum

Taxon classificationPlantaeCaryophyllalesCaryophyllaceae

(C.Y. Wu ex L.H. Zhou) Rabeler & W.L. Wagner comb. nov.

urn:lsid:ipni.org:names:77155320-1

Arenaria trichophylla C.Y. Wu ex L.H. Zhou, Acta Biol. Plateau Sin. 6: 27. 1987.

##### Type.

China: Yunnan: grassy slope, Mu-li, Hwa-to, 3900 m, 28 July 1937, F.I.B. Yunnan Exp., T.T. Yü 7428 (holotype, KUN, KUN1205449; isotype, KUN, KUN0511370).

#### Odontostemma tumengelaense

Taxon classificationPlantaeCaryophyllalesCaryophyllaceae

(L.H. Zhou) Rabeler & W.L. Wagner comb. nov.

urn:lsid:ipni.org:names:77155321-1

Arenaria tumengelaensis L.H. Zhou, Acta Phytotax. Sin. 18: 357. 1980.

##### Type.

China: Xizang: riverside grasslands, Tumengela Colliery, 5000 m, 30 July 1963, J.X. Yang 1993 (holotype, WUK, WUK0215558; isotypes, KUN [2 sheets], KUN1205455, KUN1205458).

#### Odontostemma weissianum

Taxon classificationPlantaeCaryophyllalesCaryophyllaceae

(Hand.-Mazz.) Rabeler & W.L. Wagner comb. nov.

urn:lsid:ipni.org:names:77155322-1

Arenaria weissiana Hand.-Mazz., Kaiserl. Akad. Wiss. Wien, Math.-Naturwiss. Kl., Anz. 57: 47. 1920.

##### Syntypes.

China: Yunnan: in gravelly limestone, W side of Mt. Piepun, SE of Dschungdien (Zhongdian?), 4300–4650 m, 11 August 1914, H. Handel-Mazzetti 4682 (syntype, WU, WU0043555); China: Yunnan: Mt. Yulung-schan, near Lidjiang (“Likiang”), no date given, H. Handel-Mazzetti 3657 (syntype, WU, WU0043557), same location, H. Handel-Mazzetti 3698 (syntype, WU, WU0043556; isosyntype, E, E00313715).

#### Odontostemma weissianum var. bifidum

Taxon classificationPlantaeCaryophyllalesCaryophyllaceae

(C.Y. Wu & H. Chuang) Rabeler & W.L. Wagner comb. nov.

urn:lsid:ipni.org:names:77155326-1

Arenaria weissiana var. bifida C.Y. Wu & H. Chuang in C.Y. Wu et al, Fl. Yunnan. 6: 836. 1995.

##### Type.

China: Yunnan: mountain slope, Huann-fu-ping, A-tun-tze, August 1935, C.W. Wang 68878 (holotype, KUN, KUN1205457; isotypes, A, A00235698, PE [2 sheets], PE00574388, PE00574392).

#### Odontostemma weissianum var. puberulum

Taxon classificationPlantaeCaryophyllalesCaryophyllaceae

(C.Y. Wu ex L.H. Zhou) Rabeler & W.L. Wagner comb. nov.

urn:lsid:ipni.org:names:77155327-1

Arenaria weissiana var. puberula C.Y. Wu ex L.H. Zhou, Acta Biol. Plateau Sin. 6: 34. 1987.

##### Type.

China: Sichuan: cliffs, Mu-li, Hwa-to, 3900 m, 28 July 1937, F.I.B. Yunnan Exp., T.T. Yü 7432, (holotype, KUN, KUN1205460; isotypes, KUN, KUN1205459, PE [2 sheets], PE00025036, PE00574410).

#### Odontostemma xerophilum

Taxon classificationPlantaeCaryophyllalesCaryophyllaceae

(W.W. Sm.) Rabeler & W.L. Wagner comb. nov.

urn:lsid:ipni.org:names:77155323-1

Arenaria xerophila W.W. Sm., Notes Roy. Bot. Gard. Edinburgh 11: 198. 1920.

##### Syntypes.

China: Yunnan: limy pasture and ledges of limestone cliffs, mountains in N.E. of Yangzi bend, 3050 m, 27°45'N, September 1913, G. Forrest 10998 (syntype, E, E00313694; isosyntypes, K, K000723889, KUN, KUN0511441, PE, PE00574412); China: Yunnan: open dry pasture, Atuntze valley, 28°28'N, August 1914, G. Forrest 13210 (E, not seen; isosyntypes, ISBC, not seen, PE, PE00574414).

#### Odontostemma xerophilum var. xiangchengense

Taxon classificationPlantaeCaryophyllalesCaryophyllaceae

(L.H. Zhou) Rabeler & W.L. Wagner comb. nov.

urn:lsid:ipni.org:names:77155345-1

Arenaria xiangchengensis L.H. Zhou, Acta Biol. Plateau Sin. 6: 36. 1987; Arenaria xerophila var. xiangchengensis (L.H. Zhou) C.Y. Wu in W. T. Wang et al., Vasc. Pl. Hengduan Mount. 1: 404. 1993.

##### Type.

China: Sichuan: margin of pine forest, Xiangcheng, Tungzung, 3000 m, 20 September 1937, F.I.B. Yunnan Exp., T.T. Yü 13403 (holotype, KUN, KUN0511449; isotypes, A, A00235702, PE, PE00574411).

#### Odontostemma yulongshanense

Taxon classificationPlantaeCaryophyllalesCaryophyllaceae

(L.H. Zhou ex C.Y. Wu) Rabeler & W.L. Wagner comb. nov.

urn:lsid:ipni.org:names:77155346-1

Arenaria yulongshanensis L.H. Zhou ex C.Y. Wu in W. T. Wang et al., Vasc. Pl. Hengduan Mount. 1: 410. 1993; Arenaria trichophora Franch. var. angustifolia Franch., Pl. Delavay. 95. 1889, not Arenaria angustifolia McNeill (1961).

##### Type.

China: Yunnan: limestone rocks on snowy ridges, Likiang, 4000 m, 14 Aug 1886, P.J.M. Delavay 2480 (holotype, P, P01902968; isotypes K, K000723899, P [3 sheets], P01902969, P01902970, P01902971).

#### Odontostemma yunnanense

Taxon classificationPlantaeCaryophyllalesCaryophyllaceae

(Franch.) Rabeler & W.L. Wagner comb. nov.

urn:lsid:ipni.org:names:77155324-1

Arenaria yunnanensis Franch., Bull. Soc. Bot. France 33: 431. 1886.

##### Type.

China: Yunnan: in shady rock clefts, near summit of Mt. Pengay-tze, above Houang-kia-pin, 4 Sep 1882, P.J.M. Delavay 8 (holotype, P, P01902976; isotypes, BM, BM00946351, E, E00313693, K, K000723900, KUN, KUN0511455, P [5 sheets], P01902977, P01902978, P01902980, P01902981, P01902982).

#### Odontostemma yunnanense var. caespitosum

Taxon classificationPlantaeCaryophyllalesCaryophyllaceae

(C.Y. Wu) Rabeler & W.L. Wagner comb. nov.

urn:lsid:ipni.org:names:77155328-1

Arenaria yunnanensis var. caespitosa C.Y. Wu in C.Y. Wu et al., Fl. Yunnan. 6: 835. 1995.

##### Type.

China: Yunnan: Deqen, 10 July 1940, K.M. Feng 5278 (holotype, KUN, KUN1205462; isotypes, KUN [2 sheets], KUN1205461, KUN1205464, PE, PE00574440).

#### Odontostemma zhongdianense

Taxon classificationPlantaeCaryophyllalesCaryophyllaceae

(C.Y. Wu) Rabeler & W.L. Wagner comb. nov.

urn:lsid:ipni.org:names:77155325-1

Arenaria zhongdianensis C.Y. Wu in C.Y. Wu et al., Fl. Yunnan. 6: 152, Addenda 835. 1995.

##### Type.

China: Yunnan: Zhongdian, 3300 m, 14 August 1962, Zhongdian Exp. 832 (holotype, KUN, KUN1205465; isotypes, KUN [2 sheets], KUN1205467, KUN1205468).

### Excluded taxa

Arenaria longistyla var. eugonophylla

Although appearing in Wu et al. (2001) and attributed to Fernald as published in Rhodora 21: 5. 1919, this name does not appear on that page and we have not been able to locate a place of publication.

*Arenaria microstella* C.Y. Wu in C. L. Tang, Fl. Reipubl. Popularis Sin. 26: 213. 1996.

This taxon is likely related to *Odontostemma bomiense* (Wu et al. 2001). It was described in Chinese, thus was invalidly published, and was mentioned, but not validly published, by Wu et al. (2001).

## Supplementary Material

XML Treatment for Odontostemma

XML Treatment for Odontostemma amdoense

XML Treatment for Odontostemma auricomum

XML Treatment for Odontostemma barbatum (Franch.) Sadeghian & Zarre var. hirsutissimum

XML Treatment for Odontostemma bomiense

XML Treatment for Odontostemma chamdoense

XML Treatment for Odontostemma delavayi

XML Treatment for Odontostemma dimorphitrichum

XML Treatment for Odontostemma dsharaense

XML Treatment for Odontostemma euodontum

XML Treatment for Odontostemma filipes

XML Treatment for Odontostemma fimbriatum

XML Treatment for Odontostemma giraldii

XML Treatment for Odontostemma inconspicuum

XML Treatment for Odontostemma inornatum

XML Treatment for Odontostemma ionandrum (Diels) Sadeghian & Zarre var. melanotrichum

XML Treatment for Odontostemma karakorense

XML Treatment for Odontostemma leucasterium

XML Treatment for Odontostemma littledalei

XML Treatment for Odontostemma longicaule

XML Treatment for Odontostemma longipes

XML Treatment for Odontostemma longipetiolatum

XML Treatment for Odontostemma longisetum

XML Treatment for Odontostemma longistylum

XML Treatment for Odontostemma longistylum var. pleurogynoides

XML Treatment for Odontostemma mairei

XML Treatment for Odontostemma melanandrum

XML Treatment for Odontostemma melandryiforme

XML Treatment for Odontostemma melandryoides

XML Treatment for Odontostemma membranisepalum

XML Treatment for Odontostemma minimum

XML Treatment for Odontostemma monanthum

XML Treatment for Odontostemma moniliferum

XML Treatment for Odontostemma napuligerum

XML Treatment for Odontostemma napuligerum var. monocephalum

XML Treatment for Odontostemma nigricans

XML Treatment for Odontostemma nigricans var. zhenkangense

XML Treatment for Odontostemma nivalomontanum

XML Treatment for Odontostemma omeiense

XML Treatment for Odontostemma paramelanandrum

XML Treatment for Odontostemma pharense

XML Treatment for Odontostemma polyspermum

XML Treatment for Odontostemma pseudostellaria

XML Treatment for Odontostemma quadridentatum

XML Treatment for Odontostemma reductum

XML Treatment for Odontostemma rockii

XML Treatment for Odontostemma saginoides

XML Treatment for Odontostemma salweenense

XML Treatment for Odontostemma schneiderianum

XML Treatment for Odontostemma setiferum

XML Treatment for Odontostemma spathulifolium

XML Treatment for Odontostemma szechuense

XML Treatment for Odontostemma thangoense

XML Treatment for Odontostemma trichophyllum

XML Treatment for Odontostemma tumengelaense

XML Treatment for Odontostemma weissianum

XML Treatment for Odontostemma weissianum var. bifidum

XML Treatment for Odontostemma weissianum var. puberulum

XML Treatment for Odontostemma xerophilum

XML Treatment for Odontostemma xerophilum var. xiangchengense

XML Treatment for Odontostemma yulongshanense

XML Treatment for Odontostemma yunnanense

XML Treatment for Odontostemma yunnanense var. caespitosum

XML Treatment for Odontostemma zhongdianense
